# How social media are changing pediatricians and pediatrics? – A claim for regulation

**DOI:** 10.1186/s13052-024-01822-7

**Published:** 2024-11-25

**Authors:** Saverio La Bella, Armando Di Ludovico, Niccolò Parri, Antonio Di Mauro, Antonio Corsello

**Affiliations:** 1 Department of Pediatrics, University of Chieti G. D’Annunzio, Chieti, Italy; 2 UOC Rheumatology and Autoinflammatory Diseases, IRCCS Istituto Giannina Gaslini, Genoa, Italy; 3 Department of Emergency Medicine and Trauma Center, Meyer Children’s Hospital IRCCS, Florence, Italy; 4 Pediatric Primary Care, Bari, Italy; 5 University of Milan, Milan, Italy; 6 National Association of Pediatric Residents, Padua, Italy

**Keywords:** Social media, Pediatrics, Education, Children, Healthcare, Network, Artificial intelligence

## Abstract

**Background:**

Social media has revolutionized the way healthcare professionals communicate with the public, particularly in Pediatrics. With over 5 billion users globally, platforms such as Facebook, Instagram, and TikTok have become increasingly popular even among caregivers in recent years. These channels offer unique opportunities to improve public health education, allowing pediatricians to reach a wide audience with evidence-based content.

**Main body:**

However, the risks associated with misinformation pose significant challenges to health professionals and medical organizations. In response, new recommendations for the proper use of social media in pediatric health communication should be proposed, aiming to provide a network where pediatricians can collaborate, share evidence-based information, and develop effective strategies for digital communication. With the growing use of artificial intelligence in healthcare and the rise of parental self-care practices, pediatricians must actively curate and share reliable information.

**Conclusion:**

This could serve as a new hub for ensuring that accurate, high-quality evidence-based information is disseminated, balancing the benefits of digital health advancements with the ethical responsibility of safeguarding patient care. By prioritizing professionalism, ethical communication, and technological adaptation, the aim should be to foster a more informed and health-conscious community.

## Background

The global number of social media users has reached 5.1 billion, with daily usage increased from 90 min in 2012 to 143 min today. Western Europe has one of the highest social media penetration rates, with Italy at around 72.8%. In Italy, 44.4 million internet users engage with social media monthly, a number expected to increase up to 47.6 million in 2029 [1]. A survey of over 25,000 individuals aged 16–64 found that fewer than 15% reported that social media diminished their access to information, ease of communication, or freedom of expression [1, 2]. Social media preferences vary by country and age group, with Facebook, TikTok, and Instagram being popular platforms, especially since the COVID-19 pandemic. Facebook remains the most widely used platform in Italy, with 27 million active Instagram users as of early 2024 [1]. TikTok is growing rapidly among younger demographics. This is evident as the platform had 8.9 million users in Italy in 2021and approximately 670,000 downloads in July 2024. The North American Society for Pediatric Gastroenterology, Hepatology, and Nutrition (NASPGHAN) recently released a position paper advocating for the integration of social media into medical education and academic promotion [3]. They recommend establishing a professional online presence, evaluating of digital scholarship, and using alternative metrics to assess the academic impact of social media, potentially incorporating it into academic promotion processes.

## Main text

### Challenges and opportunities in pediatrics in the post-pandemic era

The medical community has somewhat overlooked the impact of social media, despite its growing role as a major source of information for health professionals (HPs), patients, and caregivers. In pediatrics, social media offers new opportunities to enhance communication with families and colleagues, improve networking, and provide real-time access to educational content.

These platforms also allow the dissemination of accurate pediatric health information to a broad audience. However, concerns about misinformation are widespread, with percentages of users expressing scepticism about the reliability of information on social media even exceeding 70%, largely due to the prevalence of non-evidence-based content from uncertified creators and commercial interests [1, 4].

Research on pediatric educational content, such as pediatric rheumatology on TikTok, reflects these concerns. Educational videos produced by HPs, including pediatricians, physicians, physical therapists, and nurses, are of higher quality, better understood, and carry less misinformation than those made by non-HPs (patients, caregivers, influencers). Yet, HPs account for only a minority of content creators, with pediatricians representing less than 3% [5]. This suggests that increasing HP-generated content could improve the accuracy and effectiveness of health information, positively influencing caregiver knowledge and pediatric health management.

Social media can have a positive impact on pediatric health if managed properly. For instance, social media campaigns and digital reminders significantly increased COVID-19 vaccine uptake among children, compared to traditional counseling alone [6, 7]. Despite these benefits, risks such as misinformation, unverified content, and ethical concerns about privacy and professionalism pose challenges that HPs and medical organizations must address [5, 8, 9].

Pediatricians and other HPs who engage on social media should ensure their content is accurate, accessible, and adheres to high standards, maintaining trust with families, and limiting the spreading of misinformation. The rise of artificial intelligence-driven tools, such as chatbots and large language models, further complicates this landscape by promoting “self-care” medicine, through real-time but potentially unreliable health advice [10]. HPs should remain active on social platforms to provide accurate, evidence-based information and counter the risks associated with AI-related misdiagnoses and misinformation.

Leading pediatric experts have called for guidelines and regulations governing pediatric healthcare content on social media to mitigate misinformation [3, 9, 11]. A professional network of “social” pediatricians could enhance public health education by sharing best practices, developing ethical communication strategies, and fostering collaboration in pediatric knowledge dissemination. This network would prioritize evidence-based content, professional integrity, and ethical communication, countering the influence of misinformation and commercial interests. Key goals and recommendations for this network are summarized in Table 1.

**Table 1:** Key recommendations for pediatricians and pediatric residents using social media platforms

1. To clearly detail professional belonging, declaring the status of pediatricians or pediatric residency to be more trustable from patients and their families.
2. To guarantee high standards of ethics and professionalism, discussing every disclosure on content created, such as advertisement purpose, or conflicts of interest.
3. To combat misinformation, providing only evidence-based medicine, always showing the references and how to obtain more information on the topic.
4. Producing highly accurate, understandable, and actionable content, following validated scales and guidelines.
5. Sharing and promoting public health resources on important topics promoted by national and international scientific societies, such as vaccinations.

## Conclusions

Continuous feedback is essential to maintain effective strategies in addressing the growing challenges of digital health communication in pediatrics. By leveraging the power of social media, a network of pediatricians can serve as a safeguard against misinformation and unethical digital practices. With the growing role of artificial intelligence and parental self-care in healthcare, HPs must ensure their digital presence remains authoritative and indispensable. These efforts will promote a more informed, health-conscious community, providing families and children with trustworthy and accurate information, while maintaining professionalism and ethical standards in pediatric care.

## Data Availability

Not applicable.
